# RalF-Mediated Activation of Arf6 Controls Rickettsia typhi Invasion by Co-Opting Phosphoinositol Metabolism

**DOI:** 10.1128/IAI.00638-16

**Published:** 2016-11-18

**Authors:** Kristen E. Rennoll-Bankert, M. Sayeedur Rahman, Mark L. Guillotte, Stephanie S. Lehman, Magda Beier-Sexton, Joseph J. Gillespie, Abdu F. Azad

**Affiliations:** Department of Microbiology and Immunology, University of Maryland School of Medicine, Baltimore, Maryland, USA; The University of Massachusetts Medical School

## Abstract

Rickettsiae are obligate intracellular pathogens that induce their uptake into nonphagocytic cells; however, the events instigating this process are incompletely understood. Importantly, diverse Rickettsia species are predicted to utilize divergent mechanisms to colonize host cells, as nearly all adhesins and effectors involved in host cell entry are differentially encoded in diverse Rickettsia species. One particular effector, RalF, a Sec7 domain-containing protein that functions as a guanine nucleotide exchange factor of ADP-ribosylation factors (Arfs), is critical for Rickettsia typhi (typhus group rickettsiae) entry but pseudogenized or absent from spotted fever group rickettsiae. Secreted early during R. typhi infection, RalF localizes to the host plasma membrane and interacts with host ADP-ribosylation factor 6 (Arf6). Herein, we demonstrate that RalF activates Arf6, a process reliant on a conserved Glu within the RalF Sec7 domain. Furthermore, Arf6 is activated early during infection, with GTP-bound Arf6 localized to the R. typhi entry foci. The regulation of phosphatidylinositol 4-phosphate 5-kinase (PIP5K), which generates PI(4,5)P_2_, by activated Arf6 is instrumental for bacterial entry, corresponding to the requirement of PI(4,5)P_2_ for R. typhi entry. PI(3,4,5)P_3_ is then synthesized at the entry foci, followed by the accumulation of PI(3)P on the short-lived vacuole. Inhibition of phosphoinositide 3-kinases, responsible for the synthesis of PI(3,4,5)P_3_ and PI(3)P, negatively affects R. typhi infection. Collectively, these results identify RalF as the first bacterial effector to directly activate Arf6, a process that initiates alterations in phosphoinositol metabolism critical for a lineage-specific Rickettsia entry mechanism.

## INTRODUCTION

Intracellular bacteria have evolved multiple strategies to promote their entry and survival within eukaryotic host cells (1). Some intracellular pathogens, such as Yersinia pseudotuberculosis, Listeria monocytogenes, Salmonella enterica, Shigella flexneri, and Rickettsia species, induce their uptake into nonphagocytic host cells (2). Collectively, these “invasive bacteria” induce host cytoskeletal actin polymerization that results in plasma membrane (PM) rearrangement and enclosure around the pathogen, ultimately forming an intracellular vesicle or vacuole. Following entry into host cells, the vacuolar bacteria are destined for lysosomal degradation through the endolysosomal pathway, which includes early endosomes, late endosomes, and lysosomes. To disrupt this bactericidal pathway, some bacteria escape this vacuole to reside within the cytoplasm of the host cell (or within an organelle). Others modify the vacuole to prevent vacuole-lysosome fusion or customize the fused lysosome-vacuole compartment to form a replicative niche (3–5). It is well established that bacterial secreted effectors (i.e., actin nucleators, phospholipases, kinases, and receptor ligands) delicately regulate these processes (6–8). One such common target of these bacterial effectors is lipids and their metabolism (9). A class of eukaryotic membrane phosphatidylglycerides termed phosphoinositides (PIs), in particular, are key players in recruiting specific host proteins to membranes, modifying actin cytoskeleton and/or controlling maturation of intracellular compartments (10–12). Therefore, modification of PI metabolism is a significant advantage for invading pathogens.

Phosphatidylinositol 4,5-bisphosphate [PI(4,5)P_2_] has been extensively studied for its role in endocytosis, with its accumulation on membranes playing a key role in the recruitment of actin remodeling proteins, such as the Rho family of small GTPases (Rho, Rac, and Cdc42) (13). In turn, these small GTPases activate either Wiscott-Aldrich syndrome protein (WASP) or WASP family verprolin homologous proteins 1/2 (WAVE1/2), both of which bind to the cytoskeletal regulatory complex actin-related proteins 2/3 (Arp2/3) and stimulate actin polymerization (14, 15). The PI(4,5)P_2_ required to initiate this process is primarily synthesized by type I phosphatidylinositol 4-phosphate 5-kinase (PIP5K), of which there are three isoforms (α, β, and γ) (16–18). The activation of PIP5K by RhoA, Rac1, Cdc42, or ADP-ribosylation factor 6 (Arf6) must be spatiotemporally regulated to allow for transient localized accumulation of PI(4,5)P_2_, which limits actin remodeling to the site of endocytosis (19–21).

Arf6 belongs to the Arf family of small GTP-binding proteins and regulates membrane trafficking and the actin cytoskeletal network at the PM (22). In particular, it is involved in membrane trafficking during receptor-mediated endocytosis, endosomal recycling, and exocytosis of secretory granules (23–26). Like all small GTP-binding proteins, Arf6 cycles between its GTP-bound active form and GDP-bound inactive confirmation. Hydrolysis of bound GTP is mediated by GTPase-activating proteins (GAPs), while the exchange of GDP for GTP is mediated by guanine nucleotide-exchange factors (GEFs). GTP-bound Arf6 activates and regulates downstream enzymes, including PIP5K (20). Activation of Arf6 by several intracellular bacteria, including S. flexneri, S. enterica, Y. pseudotuberculosis, and Chlamydia trachomatis, is critical for their invasion (27–30). The precise mechanisms, in particular the bacterial effectors (if any), leading to Arf6 activation are unknown for all but S. flexneri, which recruits the cytohesin GEF, ARF nucleotide binding site opener (ARNO), to activate Arf6 (27).

All known species of Rickettsia are Gram-negative, obligate intracellular Alphaproteobacteria that induce their uptake into nonprofessional phagocytes, a process reliant on activation of the Arp2/3 complex and actin polymerization (31, 32). Quickly after inducing phagocytosis, bacteria escape into the host cell's cytoplasm and actively replicate. Several adhesion and cytoskeleton-binding proteins (Sca5, Adr1, Adr2, Sca4, Sca0, Sca1, Sca2, and RickA) have been identified for their role in rickettsial entry (33–39), as have several enzymes putatively involved in phagosomal escape (TlyC, PLD, Pat1, and Pat2) (40–43). Importantly, these pathogenicity factors are variably encoded across diverse Rickettsia species, indicating the likelihood of multiple strategies for rickettsial invasion of host cells (44).

Currently, the predominant knowledge of rickettsial entry is based on studies from spotted fever group (SFG) rickettsiae. For Rickettsia conorii, the surface antigen Sca5 was shown to bind host receptor Ku70, precipitating a signaling cascade that ultimately activates Arp2/3 and leads to actin polymerization, membrane rearrangement, and bacterial invasion (45, 46). Disruption of these signaling pathways identified Cdc42, phosphatidylinositol 3-kinases (PI3Ks), c-Src, and other protein tyrosine kinase activities as critical factors for R. conorii invasion of nonphagocytic cells (31). The strict conservation of *sca5* in all Rickettsia genomes implies that this invasion strategy is highly conserved. However, for R. parkeri, knockdown or inhibition of individual Arp2/3 complex activators revealed a modest decrease in invasion compared to depletion or inhibition of the Arp2/3 complex, indicating that either redundant pathways converge to activate Arp2/3 or a bacterial effector activates Arp2/3 (47). One effector that could potentially fulfill this role is the Arp2/3-activating protein, RickA, which is encoded in most SFG rickettsia genomes and has been shown to be critical for early Rickettsia parkeri motility (32). However, the lack of *rickA* genes in other Rickettsia species, particularly those of typhus group (TG) rickettsiae, indicates that different Rickettsia species utilize divergent strategies to induce cytoskeletal rearrangement during host cell invasion.

We recently proposed a role for another rickettsial effector, RalF, in actin rearrangement and bacterial entry (48). Importantly, the function of RalF is not complementary to RickA, provided that some Rickettsia species (e.g., *R*. bellii, R. felis, and R. akari) encode both proteins. Furthermore, a distinct molecular mechanism of RalF is apparent, as the protein contains a eukaryotic Sec7 domain that functions as an Arf-GEF (49, 50). Known only from species of Legionella and Rickettsia (51), the reported functions for RalF differ markedly across these divergent pathogens (48, 52, 53). Legionella RalF (RalF_L_), which recruits and activates host Arf1 at the Legionella-containing vacuole (49, 54), was shown to localize to the endoplasmic reticulum, where it likely plays a role in intercepting host secretory vesicles (52, 53). In contrast, RalF of several diverse Rickettsia species was shown to localize to the host PM (48). For R. typhi RalF (RalF_Rt_), PM localization was PI(4,5)P_2_ dependent, and an interaction with host Arf6 was found to be critical for R. typhi infection (48). Furthermore, RalF_Rt_ inactivation via antibody blocking significantly decreased R. typhi infection, bolstering the role for this effector as a lineage-specific rickettsial invasin.

As Arf6 is known to play a role in actin remodeling via its activation of PIP5K and subsequent synthesis of PI(4,5)P_2_, we focused our present investigation into the role of RalF_Rt_-activated Arf6 and subsequent PI metabolism during invasion. Herein, we identify RalF as the first bacterial effector directly capable of activating Arf6 to initiate changes in PI metabolism critical for rickettsial entry.

## MATERIALS AND METHODS

### Bacterial strains, cell culture, and infection.

Vero76 (African green monkey kidney, ATCC, RL-1587) and HeLa (ATCC, CCL-2) cells were maintained in minimal Dulbecco's modified Eagle's medium (DMEM; with 4.5 g/liter glucose and 480 l-glutamine [Mediatech, Inc.]) supplemented with 10% heat-inactivated fetal bovine serum (FBS) at 37°C with 5% CO_2_. R. typhi strain Wilmington (ATCC, VR-144) was propagated in Vero76 cells grown in DMEM supplemented with 5% heat-inactivated fetal bovine serum at 34°C with 5% CO_2_. Rickettsiae were purified as previously described (55). For host cell infections, R. typhi was used at a multiplicity of infection (MOI) of ∼100:1. Prior to infection, cells were washed with DMEM with 5% FBS. Where indicated, cells were pretreated for 2 h with 100 nM wortmannin (Sigma), 5 mM LY294002 (Sigma), or an equal volume of the solvent dimethyl sulfoxide (DMSO) (Sigma); inhibitors were maintained in the medium throughout infections.

### Mammalian expression plasmids.

A plasmid coding for the Arf binding domain of ARHGAP tagged with enhanced green fluorescent protein (EGFP; pEGFP-ARHGAP10 Arf-BD) was a generous gift from Philippe Chavrier (Institut Curie, Paris, France) (56). The hemagglutinin (HA)-tagged mouse PIP5Kβ wild type and catalytically dead mutant (D227A) in the pcDNA vector were kind gifts from Ralph Isberg (Tufts University School of Medicine, Boston, MA) (29). pEGFP-2xFYVE was a kind gift from George Banting (University of Bristol, Bristol, United Kingdom) (57). The plasmid coding for green fluorescent protein (GFP)-tagged human PIP5K gamma 90 (GFP-PIPK1 gamma 90) was a generous gift from Pietro De Camilli (Yale School of Medicine, New Haven, CT [Addgene plasmid 22299]) (58). pcDNA3-AKT-PH-GFP was a kind gift from Craig Montell (University of California, Santa Barbara, CA [Addgene plasmid 18836]) (59). Plasmid pEYFP-RalF_Rt_ was previously described (48). pEYFP-RalF_Rt E100A_ was generated using the QuikChange Lightning multisite-directed mutagenesis kit (Agilent Technologies). Similarly, all Arf6 mutant plasmids were generated using the QuikChange Lightning multisite-directed mutagenesis kit (Agilent Technologies) with pcDNA3-mRFP-Arf6, a kind gift from Vassilis Koronakis (University of Cambridge, Cambridge, United Kingdom) (28), as a template. Primers used for mutagenesis are listed in Table 1.

**TABLE 1:** Primers used for site-directed mutagenesis

Primer	Sequence (5′ to 3′)^*a*^
RalF_Rt E100A_	CATTTAAATTACCGGGCGCCGCTCAAAAAATCGATAGG
Arf6_T27N_	CGCGGCCGGCAAGAACACAATCCTGTAC
Arf6_N48I_	CCACTGTGGGTTTCATCGTGGAGACGGTGAC
Arf6_N122I_	CCTCATCTTCGCCATCAAGCAGGACCTGC

a Underlined letters indicate a mutated codon.

### Arf6 activation assay.

Arf6 or Arf1 activation was measured using the G-LISA Arf6 Activation Assay Biochem kit or the G-LISA Arf1 Activation Assay Biochem kit (Cytoskeleton). Six-well plates seeded with HeLa cells were infected with R. typhi for 30 min. Each well was washed with 3 ml ice-cold phosphate-buffered saline (PBS) and lysed in 100 μl lysis buffer per the manufacturer's recommendations. Lysates from three wells were combined for each sample. T-75 flasks seeded with HeLa cells were transfected with 24 μg plasmid (pEYFP-C1, pEYFP-RalF_Rt_, or pEYFP-RalF_Rt E100A_) using Lipofectamine 2000 (Thermo Fisher Scientific) per the manufacturer's recommendations. Twenty-four hours posttransfection, cells were washed with 8 ml ice-cold PBS and lysed in 400 μl lysis buffer. The protein concentration was determined using the Precision Red protein assay reagent (Cytoskeleton), and a concentration of 1 mg/ml of lysate was used for the assays. Expression of enhanced yellow fluorescent protein (EYFP)-RalF_Rt_ and EYFP-RalF_Rt E100A_ relative to GAPDH (glyceraldehyde-3-phosphate dehydrogenase) expression was determined by protein immunoblotting with anti-GFP mouse antibody (Pierce) and anti-GAPDH mouse antibody (Abcam). Densitometry analysis was performed with ImageJ (NIH).

The G-LISA kits contain a 96-well plate with an Arf6-GTP or Arf1-GTP binding protein cross-linked to the wells. Active GTP-bound Arf in cell lysates is pulled down by the Arf-GTP binding protein and is detected with an Arf6- or Arf1-specific antibody. Signal is detected with horseradish peroxidase (HRP) detection reagents, and absorbance was measured at 490 nm by a plate reader (FLUOstar Omega plate reader; BMG Labtech). All experiments were repeated in triplicate with four technical replicates each, and a Student's two-sided *t* test or one-way analysis of variance (ANOVA) and Dunnett's multiple-comparison test were performed to determine statistical significance compared to untreated or EYFP-transfected HeLa cells.

### Immunofluorescence.

Eight-well chamber slides were seeded with HeLa cells, which were then transfected with 1 μg plasmid per well using Lipofectamine 2000 (Thermo Fisher Scientific) per the manufacturer's recommendations. Five to 6 h posttransfection, cells were infected with R. typhi, described above, for 5 to 15 min or 2 h. Cells were washed three times with phosphate-buffered saline (PBS) and fixed with 4% paraformaldehyde (PFA) for 20 min at room temperature. Cells were washed three times with PBS and permeabilized in blocking buffer (0.2% saponin and 5% FBS in PBS) for 30 min. Cells were incubated for 1 h with primary antibodies rat anti-R. typhi serum (1:500) and mouse anti-HA epitope tag (2-2.2.14) (1:1,000 [Pierce]) diluted in antibody dilution buffer (0.3% saponin in PBS). Cells were then washed with PBS and incubated for 1 h with anti-mouse and anti-rat Alexa Fluor 594 or Alexa Fluor 488 secondary antibodies (Thermo Fisher Scientific) diluted 1:1,500 in antibody dilution buffer. For PI3K inhibitor assays, cell membranes were stained with wheat germ agglutinin-Alexa Fluor 594 conjugate (Thermo Fisher Scientific) according to the manufacturer's protocol. Finally, cells were washed three times with PBS and mounted using ProLong Gold antifade mounting medium with DAPI (4′,6-diamidino-2-phenylindole [Thermo Fisher Scientific]). For confocal microscopy, cells were viewed under a Zeiss LSM510 Meta confocal microscope (University of Maryland Baltimore Confocal Core Facility). For conventional fluorescence microscopy, a Nikon Eclipse E600 fluorescence microscope with a Q Imaging Retiga 2000R camera was used to capture images with QCapture Pro software. Fluorescence intensity for a given area surrounding a single bacterium was calculated for 5 to 10 bacteria using ImageJ software (NIH). The number of bacteria per cell was counted for 100 cells per well in triplicate. Experiments were repeated 2 to 3 times, and Student's two-sided *t* test or one-way ANOVA and Dunnett's multiple-comparison test were performed to determine statistical significance.

## RESULTS

### R. typhi RalF activates Arf6.

**FIG 1:**
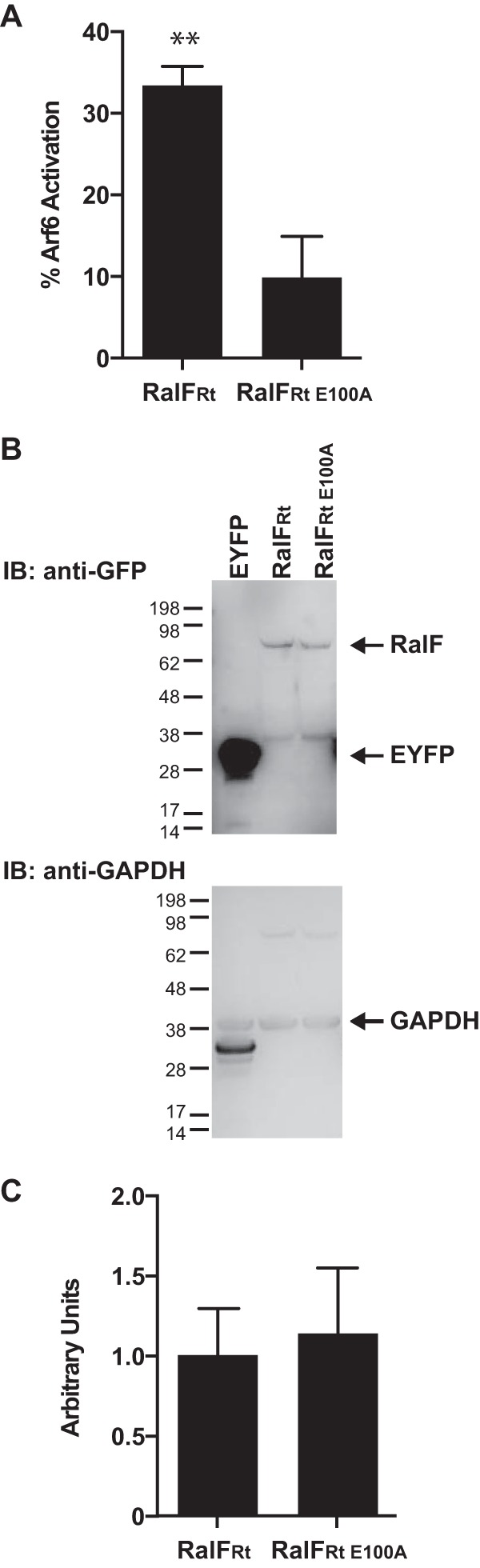
RalF_Rt_ activates Arf6. (A) HeLa cells overexpressing EYFP, EYFP-RalF_Rt_, or EYFP-RalF_Rt E100A_ were assayed for Arf6 activation using the G-LISA Arf6 Activation Assay Biochem kit (Cytoskeleton). The percentage of Arf6 activation calculated relative to cells transfected with EYFP only is shown. Error bars represent means ± standard errors of the means (SEM) from three independent experiments. EYFP-RalF_Rt_, but not EYFP-RalF_Rt E100A_, significantly increased Arf6 activation compared to the level with EYFP alone. **, *P* < 0.01 by one-way ANOVA and Dunnett's multiple-comparison test. (B) Protein immunoblot (IB) of representative lysates assayed for Arf6 activation. HeLa cells ectopically expressing EYFP, EYFP-RalF_Rt_, or EYFP-RalF_Rt E100A_ were lysed, and equal concentrations of cellular lysate were analyzed by protein immunoblotting using an anti-GFP antibody and reprobed with anti-GAPDH antibody as indicated. The additional bands migrating near EYFP for EYFP-RalF_Rt_ and EYFP-RalF_Rt E100A_ are potentially cleaved EYFP or a nonspecific binding product. (C) Densitometry analysis using ImageJ software (NIH) was performed to ensure equal quantities of EYFP-RalF_Rt_ and EYFP-RalF_Rt E100A_ relative to GAPDH in the lysates used for the Arf6 activation assays (A). The means ± SEM from three independent experiments are plotted. No statistically significant difference in densitometry was detected using a Student's two-sided *t* test (*n* = 3).

To further explore the dynamics of the RalF_Rt_-Arf6 interaction that is critical for R. typhi infection (48), we set out to resolve the downstream effects of this interaction, beginning with assays to determine the ability of RalF_Rt_ to activate Arf6. Cellular lysates from HeLa cells transfected with either pEYFP-C1 (a background control for Arf6 activation) or pEYFP-RalF_Rt_ for 24 h were tested for Arf6 activation. Arf6 was significantly activated with an approximate 30% increase in GTP-bound Arf6 in HeLa cells ectopically expressing EYFP-RalF_Rt_ compared to the level in cells expressing EYFP (Fig. 1A). These data indicate that RalF_Rt_ is a functional Arf-GEF with the ability to exchange GDP for GTP on host Arf6 molecules.

The Arf-GEF activities of RalF_L_ and RalF_Rp_ are reliant on the highly conserved Sec7 domain, as mutations of the active site Glu significantly inhibit Arf activation (52). We therefore sought to determine if the conserved Glu within the RalF_Rt_ Sec7 domain is critical for its Arf-GEF activity. Using site-directed mutagenesis, we generated the pEYFP-RalF_Rt E100A_ plasmid, in which the active site Glu was mutated to Ala. HeLa cells were transfected for 24 h, and expression of EYFP-RalF_Rt E100A_ was assessed using protein immunoblot analysis; a representative blot is shown in Fig. 1B. Densitometry was performed to ensure equal quantities of EYFP-RalF_Rt_ and EYFP-RalF_Rt E100A_ relative to the GAPDH control in the cellular lysates used to assay for Arf6 activation (Fig. 1C). As predicted, overexpression of EYFP-RalF_Rt E100A_ did not significantly increase Arf6 activation compared to that in cells expressing EYFP (Fig. 1A), further illustrating the essentiality of the Sec7 active site Glu for efficient GDP/GTP exchange on Arf proteins.

### R. typhi infection activates Arf6.

**FIG 2:**
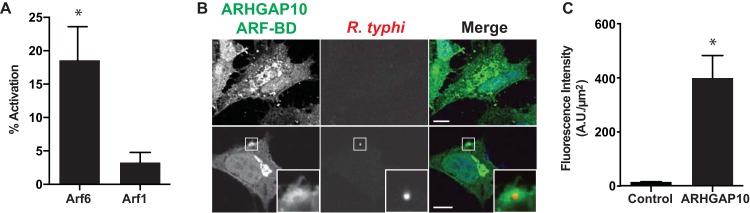
Arf6 is activated and localized to R. typhi entry foci. (A) Arf6 but not Arf1 is activated early during R. typhi infection. HeLa cells incubated with R. typhi for 30 min were assayed for endogenous Arf6 and Arf1 activation using G-LISA Arf6 and Arf1 Activation Assay Biochem kits (Cytoskeleton). The percentage of activation of Arf6 or Arf1 was calculated in HeLa cells 30 min postinfection with R. typhi (MOI, 100:1) compared to that in uninfected HeLa cells. Arf6, but not Arf1, was significantly activated by R. typhi infection compared to the level in uninfected controls. Error bars represent means ± SEM from three independent experiments. *, *P* < 0.05 by Student's two-sided *t* test. (B) GTP-bound Arf6 localizes to R. typhi entry foci. HeLa cells overexpressing the ARF-binding domain of ARHGAP10 (an Arf6 GTP-bound biosensor) fused to GFP were exposed to R. typhi (MOI, 100:1) for 15 min (bottom panel) or mock treated (top panel). Cells were fixed with 4% paraformaldehyde, and R. typhi was detected with anti-R. typhi Ab (red). DAPI (blue) is shown in the merged image. Boxed regions are enlarged to show detail. Scale bar, 10 μm. (C) Quantification of ARHGAP10 Arf-BD localization at the R. typhi entry foci. Cells expressing GFP-tagged ARHGAP10 Arf-BD or GFP alone were infected with R. typhi and processed as described for panel B. Fluorescence immediately surrounding R. typhi was measured using ImageJ (NIH) and expressed as arbitrary units (A.U.) per square micrometer. The means ± SEM of 10 to 15 bacteria from two independent experiments are plotted. *, *P* < 0.05 by Student's two-sided *t* test.

The *in vitro* Arf6 activation by RalF_Rt_, coupled with our prior demonstration that R. typhi expresses and secretes this effector early during host cell invasion (48), led us to determine if Arf6 is activated during R. typhi entry. Arf6 activation increased ∼20% during R. typhi infection compared to the level in uninfected controls (Fig. 2A). As a negative control, we assayed for Arf1 activation, as Arf1 does not colocalize with RalF_Rt_ (48). Indeed, Arf1 was not activated during R. typhi entry compared to the uninfected control. These data suggest endogenous Arf6 is specifically activated early during R. typhi infection.

We previously showed that ectopically expressed Arf6 localizes to the R. typhi entry foci (48). Here we sought to determine if the recruited Arf6 is activated. To detect endogenous activated Arf6 localization, we used an Arf6-GTP biosensor. The Arf binding domain of ARHGAP10 (ARHGAP10 Arf-BD) has been shown to bind activated Arf6, as well as activated Arf1 (56). We ectopically expressed the ARHGAP10 Arf-BD with a GFP tag in HeLa cells for 6 h followed by infection with R. typhi. Cells were fixed 15 min postinfection, and R. typhi was detected with rat anti-R. typhi serum followed by anti-rat Alexa Fluor 594 antibody. In uninfected HeLa cells, the ARHGAP10 Arf-BD localized primarily within the host cytoplasm. Upon R. typhi infection, the ARHGAP10 Arf-BD localized to the PM at the site of R. typhi entry (Fig. 2B and C). Furthermore, the ARHGAP10 Arf-BD localization resembled that of Arf6 (48), suggesting that the Arf6 recruited to the R. typhi entry foci is activated.

### Arf6 mutants decrease R. typhi infection.

**FIG 3:**
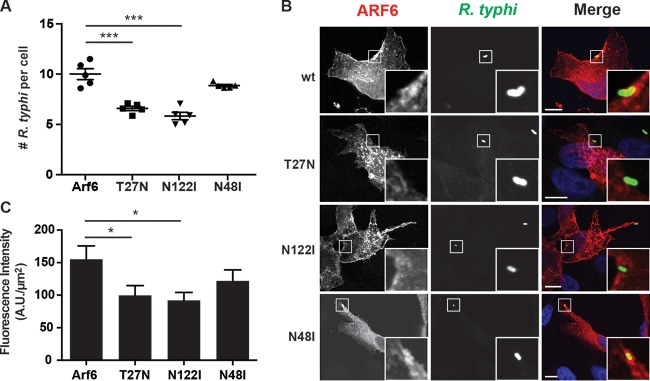
Inhibition of Arf6 impairs R. typhi infection. (A) Arf6_T27N_ and Arf6_N122I_ decrease R. typhi infection. HeLa cells transiently expressing the mRFP-Arf6 wt or T27N, N122I, or N48I mutant were incubated with partially purified R. typhi (MOI, 100:1) for 2 h at 34°C. The cells were fixed with 4% PFA, and R. typhi was detected with rat anti-R. typhi serum and anti-rat Alexa Fluor 488 antibody. The number of R. typhi bacteria per cell was counted for 100 cells per well. Means ± SEM from five wells of two independent experiments are plotted. ***, *P* < 0.0001 by one-way ANOVA and Dunnett's multiple-comparison test. (B) Arf6_T27N_ and Arf6_N122I_ do not localize to R. typhi entry foci. HeLa cells were treated as described in panel A, except the incubation time was 15 min. The cells were fixed with 4% PFA, and R. typhi was detected with rat anti-R. typhi serum and anti-rat Alexa Fluor 488 antibody (green). DAPI (blue) is shown in the merged image. Boxed regions are enlarged to show detail. Scale bars, 10 μm. (C) Quantification of Arf6 localization at the R. typhi entry foci. mRFP-tagged Arf6 wt- or T27N, N122I, or N48I mutant-expressing cells were infected with R. typhi and processed as described for panel B. Fluorescence immediately surrounding R. typhi was measured using ImageJ (NIH) and expressed as arbitrary units (A.U.) per square micrometer. The means ± SEM of 10 to 15 bacteria from two independent experiments are plotted. *, *P* < 0.05 by one-way ANOVA and Dunnett's multiple-comparison test.

Arf6 regulates the lipid-modifying enzymes phospholipase D (PLD) and PIP5K. Arf6 activation of host PLD, an enzyme that catalyzes the hydrolysis of phosphatidylcholine to generate phosphatidic acid, is critical for membrane recycling, changes in actin cytoskeleton, cell migration, and exocytosis (26, 60–62). Activation of PIP5K by Arf6 leads to the accumulation of PI(4,5)P_2_, which in turn recruits actin capping and actin binding proteins to promote actin remodeling and endocytosis (20, 22, 63). This activation of PLD and PIP5K relies on the ability of Arf6 to bind GTP, as GDP-bound and nucleotide-free Arf6 dominant-negative mutants (T27N and N122I, respectively) are unable to recruit and activate both PLD and PIP5K (22). As knockdown of Arf6 significantly decreases R. typhi infection (48), we further proceeded to determine if the activation of PLD and PIP5K via Arf6 is critical for R. typhi infection. HeLa cells transfected with Arf6_T27N_ or Arf6_N122I_ caused a 34% or 41%, respectively, decrease in the number of R. typhi organisms per cell compared to that in the wild-type Arf6-transfected cells (Fig. 3A). Additionally, the localization pattern of these mutants differs from that of wild-type Arf6. Whereas wild-type Arf6 accumulates in the PM at the site of R. typhi entry, both Arf6 mutants form intracellular vesicles that do not localize to the R. typhi entry foci (Fig. 3B and C).

To specifically determine whether Arf6 activation of PLD is critical for R. typhi invasion, HeLa cells ectopically expressing Arf6_N48I_ were exposed to R. typhi. Arf6_N48I_ lacks the ability to stimulate PLD, yet is able to activate PIP5K (26). Expression of Arf6_N48I_ did not impair R. typhi infection and was localized to the R. typhi entry foci, suggesting that regulation of PLD by Arf6 is not required for R. typhi entry (Fig. 3). Collectively, these data suggest Arf6 regulation of PIP5K is important for R. typhi infection.

### PIP5K recruitment to R. typhi entry foci.

**FIG 4:**
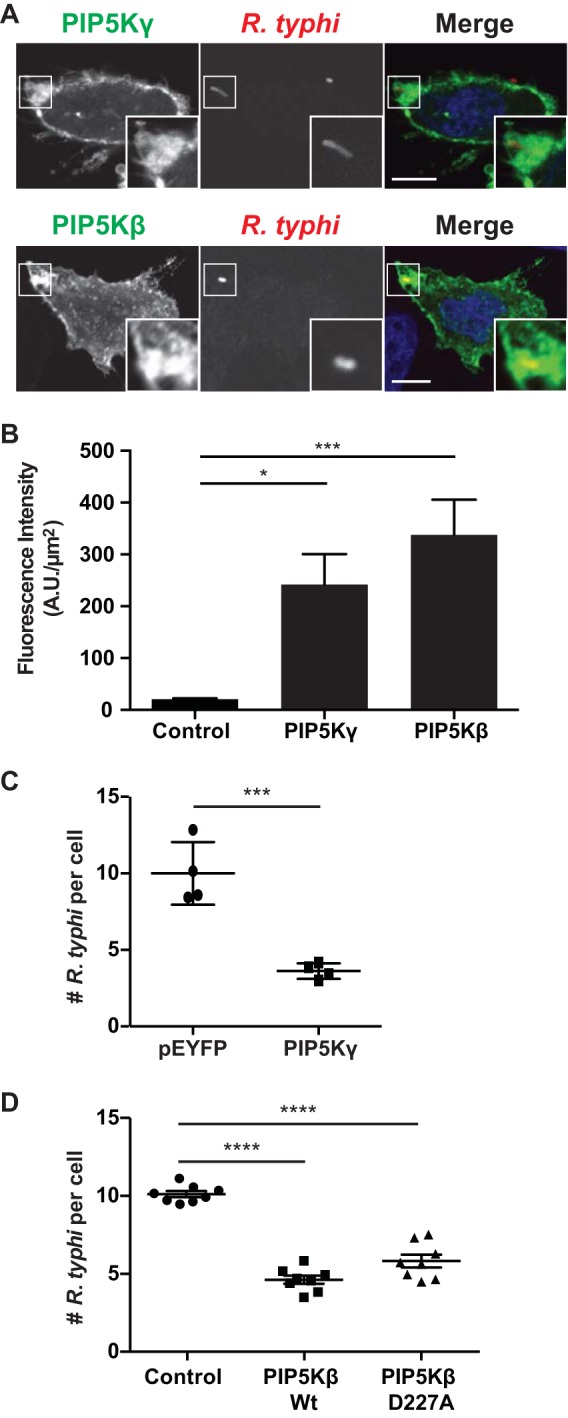
PIP5K is recruited to R. typhi entry foci. (A) HeLa cells overexpressing GFP-PIP5Kγ (top) or HA-PIP5Kβ (bottom) were incubated with R. typhi (MOI, 100:1) for 15 min. Cells were fixed with 4% PFA, and R. typhi cells were detected with rat anti-R. typhi serum and anti-rat Alexa Fluor 594 (red). HA-tagged PIP5Kβ was detected with a mouse anti-HA antibody followed by anti-mouse Alexa Fluor 488 antibody (green). DAPI (blue) is shown in the merged image. Boxed regions are enlarged to show detail. Scale bars, 10 μm. (B) Quantification of PIP5K localization at the R. typhi entry foci. GFP-, GFP-PIP5Kγ-, or HA-PIP5Kβ-expressing cells were infected with R. typhi and processed as described for panel A. Fluorescence immediately surrounding R. typhi was measured using ImageJ (NIH) and expressed as arbitrary units (A.U.) per square micrometer. The means ± SEM of 10 to 15 bacteria from two independent experiments are plotted. *, *P* < 0.05, and ***, *P* < 0.0001, by one-way ANOVA and Dunnett's multiple-comparison test. (C) Overexpression of PIP5Kγ decreases R. typhi infection. HeLa cells expressing GFP alone or GFP-PIP5Kγ were incubated with R. typhi (MOI, 100:1) for 2 h. Cells were fixed with 4% PFA, and R. typhi was detected with rat anti-R. typhi serum and anti-rat Alexa Fluor 594. The number of R. typhi bacteria per cell for 100 cells was calculated for four independent experiments, and means ± SEM are plotted. ***, *P* < 0.001 by Student's two-sided *t* test. (D) Overexpression of wild-type and catalytically dead PIP5Kβ decreases R. typhi infection. HeLa cells expressing the HA-tagged PIP5Kβ wild-type (wt) or catalytically dead mutant (D227A) were exposed to R. typhi (MOI, 100:1) for 2 h. Cells were fixed with 4% PFA, R. typhi bacteria were detected with rat anti-R. typhi serum, and HA-tagged PIP5Kβ was detected with mouse anti-HA antibody. The number of R. typhi bacteria per cell for 100 cells in four different wells per condition was calculated for three independent experiments, and means ± SEM are plotted. ****, *P* < 0.0001 by one-way ANOVA and Dunnett's multiple-comparison test.

To further determine the role of PIP5K in R. typhi infection, we monitored PIP5K subcellular localization during R. typhi invasion. HeLa cells transfected with either the β or γ isoform of PIP5K were exposed to R. typhi for 15 min. Both isoforms were recruited to the PM at the site of R. typhi entry (Fig. 4A and B). Previously, it was shown that overexpression of PIP5Kα and -β decreased Y. pseudotuberculosis infection due to the overproduction of PI(4,5)P_2_, which antagonized vacuole formation by preventing vacuole scission from the plasma membrane (64). Similarly, overexpression of either PIP5Kβ or PIP5Kγ decreased the number of R. typhi bacteria per cell by 64% and 52%, respectively, compared to the levels in cells transformed with the respective control plasmids (Fig. 4C and D). Furthermore, kinase-dead PIP5K isoforms, which are unable to synthesize PI(4,5)P_2_, interfere with endocytosis (65, 66). We quantified R. typhi infection in HeLa cells overexpressing kinase-dead PIP5Kβ (D227A) and showed a 48% decrease in the number of R. typhi cells per cell (Fig. 4D). Altogether, these data demonstrate that the overproduction of PI(4,5)P_2_ by PIP5K and further the lack of PI(4,5)P_2_ production by kinase-dead PIP5K antagonize R. typhi entry, suggesting there is a critical threshold of PI(4,5)P_2_ required for bacterial entry.

### Phosphoinositol metabolism is critical for R. typhi entry.

**FIG 5:**
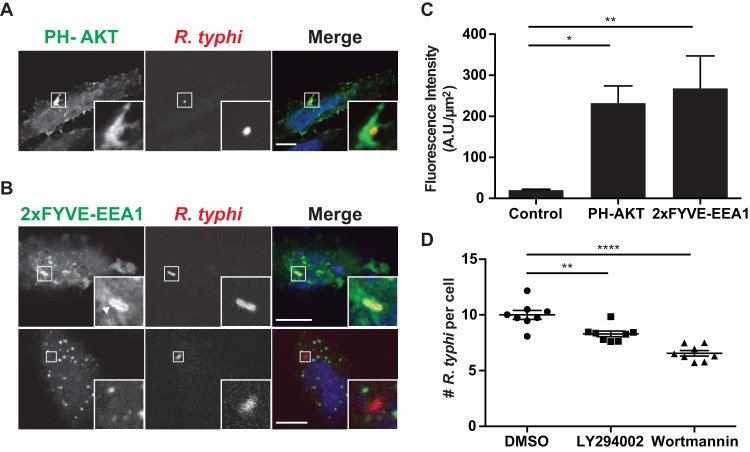
Recruitment of PI(3,4,5)P3 and PI(3)P to R. typhi entry foci. HeLa cells overexpressing (A) the pleckstrin homology (PH) domain of RAC-alpha serine/threonine-protein kinase (AKT), a PI(3,4,5)P_3_ and PI(3,4)P_2_ biosensor (71), or (B) the FYVE domain of early endosome antigen 1 (EEA1), a PI(3)P biosensor (71), were incubated with R. typhi (MOI, 100:1) for 5 min (top panel) or 15 min (bottom panel). Cells were fixed with 4% PFA, and R. typhi was detected with rat anti-R. typhi antibody and anti-rat Alexa Fluor 594 antibody (red). DAPI (blue) is shown in the merged image. Boxed regions are enlarged to show detail. Scale bars, 10 μm. (C) Quantification of PH-AKT and 2× FYVE-EEA1 localization at the R. typhi entry foci. GFP-, PH-AKT-, and 2 × FYVE-EEA1-expressing cells were infected with R. typhi and processed as described for panels A and B. Fluorescence immediately surrounding R. typhi was measured using ImageJ (NIH) and expressed as arbitrary units (A.U.) per μm^2^. The means ± SEM of 10 to 15 bacteria from two independent experiments are plotted. *, *P* < 0.05, and **, *P* < 0.001, by one-way ANOVA and Dunnett's multiple-comparison test. (D) Inhibition of PI3K decreases R. typhi infection. HeLa cells pretreated for 2 h with the PI3K inhibitor LY294002 (5 mM) or wortmannin (100 nM) were infected with R. typhi (MOI, 100:1). Cells were fixed with 4% PFA, and R. typhi was detected with rat anti-R. typhi serum and anti-rat Alexa Fluor 488 antibody and cell membrane stained with wheat germ agglutinin-Alexa Fluor 594 conjugate. The number of R. typhi bacteria per cell for 100 cells in four different wells per condition was calculated for three independent experiments, and means ± SEM are plotted. **, *P* < 0.01, and ****, *P* < 0.0001, by one-way ANOVA and Dunnett's multiple-comparison test.

Downstream of PIP5K-mediated synthesis of PI(4,5)P_2_, class I PI3K phosphorylates PI(4,5)P_2_ to yield PI(3,4,5)P_3_. This conversion to PI(3,4,5)P_3_ is critical for vacuole sealing and scission from the PM (11, 64). Generation of PI(3)P by class III PI3K (Vps34) is then critical for maturation of the phagosome (11). We first confirmed that class I PI3K is active at the sites of invasion using the pleckstrin homology (PH) domain of RAC-alpha serine/threonine-protein kinase (AKT) fused to GFP (PH-AKT), a probe for PI(3,4,5)P_3_ and/or PI(3,4)P_2_. HeLa cells transfected with PH-AKT were exposed to R. typhi (MOI, 100:1), and distribution of the probe was monitored by confocal microscopy. PH-AKT accumulated on the PM at the R. typhi entry foci (Fig. 5A and C). Furthermore, we monitored the generation of PI(3)P using the FYVE domain of early endosome antigen 1 (EEA1), which binds PI(3)P, in duplicate as a sensor. During early infection (i.e., bacteria localized near the PM, 5 min), the PI(3)P biosensor completely surrounded the bacterium (Fig. 5B and C). However, later in infection (15 min), the PI(3)P biosensor showed more punctate structures surrounding the bacterium (Fig. 5B), indicating lysis of the vacuolar membrane by R. typhi to escape into the host cytosol.

Finally, to determine the role of PI3Ks during R. typhi infection, we pretreated HeLa cells with two general PI3K inhibitors, LY294002 and wortmannin. Both inhibitors significantly decreased R. typhi infection; however, wortmannin, a more potent inhibitor, had a greater effect on R. typhi infection (Fig. 5D). These data suggest that PI metabolism is critical for R. typhi infection.

## DISCUSSION

PI metabolism plays a key role in the regulation of receptor-mediated signal transduction, actin remodeling, and membrane trafficking in eukaryotic cells. Small GTPases recruit and activate kinases, phosphatases, or phospholipases that modify PIs, allowing for rapid changes in membrane dynamics (67). Entry into a host cell is a critical step in the life cycle of intracellular pathogens and requires actin cytoskeleton and PM rearrangement, which is in part regulated by PI metabolism. Therefore, exploitation of PI metabolism and the modification of PI cellular signaling cascades are common strategies utilized by intracellular pathogens for invasion of eukaryotic cells. Here, we demonstrate that the co-option of PI metabolism via the activation of Arf6 and its downstream effector PIP5K is critical for R. typhi invasion.

**FIG 6:**
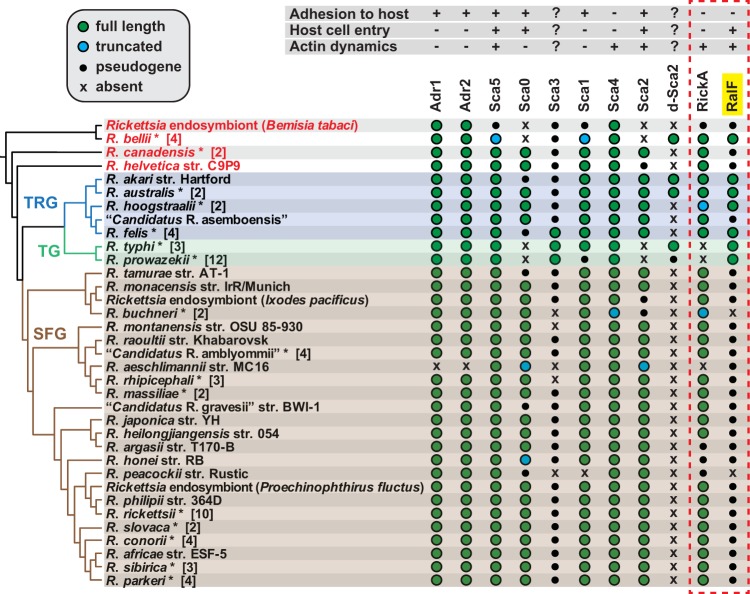
Phylogenomic analysis of Rickettsia proteins implicated in host cell invasion. The phylogeny at left, which includes 82 Rickettsia genomes, was estimated as previously described (44, 72). The inset at the top left describes gene characteristics: green, full length; blue, truncated; black, pseudogene; x, absent. Taxonomic groups (73) are as follows: TRG, transitional group rickettsiae; TG, typhus group rickettsiae; SFG, spotted fever group rickettsiae. Red taxa depict ancestral lineages. Taxa with asterisks are a composite of multiple genomes from the same species, with numbers in brackets indicating the total number of genomes analyzed. In some cases where different strains show variation for a particular gene, the characteristics of the gene from the better-quality genome were selected. Note that some composite taxa include genomes from attenuated strains (e.g., R. prowazekii strain Madrid E and R. rickettsii strain Iowa) that contain different genetic profiles than those listed. The dashed red box distinguishes the two rickettsial effectors from the nine adhesins, with RalF highlighted in yellow.

The predominant knowledge base of Rickettsia entry mechanism is currently based on studies using species of SFG rickettsiae. Importantly, nearly all of the adhesins and effectors that are either characterized or predicted to be involved in the host cell entry are differentially encoded across rickettsial lineages (Fig. 6), suggesting that diverse Rickettsia species utilize divergent mechanisms to colonize host cells. RalF is one such effector, being encoded by all species of TG and most transitional group rickettsiae, as well as the early-branching species R. bellii, but pseudogenized or absent in all species of SFG rickettsiae and other early-branching species (Fig. 6). Remarkably, analysis of host subcellular localization patterns of RalF from diverse rickettsial species (R. typhi, R. felis, and R. bellii) revealed colocalization with Arf1 at perinuclear regions for R. bellii RalF, which starkly contrasts the colocalization with Arf6 at the PM for RalF_Rt_ (48). This potential for divergent utilization of Arf-GEF activities, in conjunction with the patchy genomic distribution of the only other Rickettsia effector predicted to directly associate with the host cytoskeletal network during invasion (RickA), illuminates a quandary for identification of a universal Rickettsia entry mechanism (Fig. 6). Considering our recent findings that RalF_Rt_ and Arf6 were both critical for R. typhi infection, our goal herein was to further characterize the role of Arf6 and the downstream effects of Arf6 activation by RalF_Rt_ during R. typhi entry, keeping in mind that such work would potentially identify a novel mechanism of rickettsial entry limited to certain Rickettsia species.

Arf6 activation by some intracellular pathogens (e.g., species of Salmonella, Yersinia, Chlamydia, and Shigella) is known to induce actin remodeling and facilitate bacterial entry (27–30). Here we demonstrate that RalF_Rt_ activates Arf6, and this activation depended on the conserved Glu within its Sec7 domain. Furthermore, Arf6 (but not Arf1) was activated early during R. typhi infection and recruited to entry foci. Because we previously demonstrated that knockdown of Arf6 significantly limited R. typhi infection (48), we proceeded to further determine the mechanism by which activated Arf6 leads to R. typhi invasion.

Arf6 is involved in endocytosis via the recruitment and activation of PLD and PIP5K, which produce phosphatidic acid and PI(4,5)P_2_, respectively, which in turn modulate vesicular trafficking and actin polymerization (20, 68, 69). Our data demonstrate that PLD does not play a major role in R. typhi infection, as expression of the N48I Arf6 mutant, which is incapable of stimulating PLD activity, did not affect bacterial entry. Furthermore, Arf6_N48I_ was still recruited to the R. typhi entry foci. Conversely, overexpression of Arf6_T27N_ and Arf6_N122I_ mutants, which are incapable of activating PLD as well as PIP5K, decreased R. typhi infection, and these mutants were not recruited to the R. typhi entry foci. Collectively, these data suggest that Arf6 activation of PIP5K, but not PLD, plays a role in R. typhi invasion.

PI(4,5)P_2_, the product of PIP5K, accumulates at entry foci and is critical for R. typhi invasion, as treatment of cells with ionomycin in the presence of calcium significantly decreases R. typhi infection (48). Recruitment and activation of PIP5K by Arf6 could explain the accumulation of PI(4,5)P_2_ early during R. typhi infection. The two PIP5K isoforms assayed (β and γ) localized to R. typhi entry foci. Additionally, a balance of PI(4,5)P_2_ metabolism needs to be maintained during R. typhi entry, as overexpression of wild-type PIP5K and expression of catalytically dead PIP5Kβ decreased infection. PI(4,5)P_2_ at the PM allows for the proper orientation, activation, and coalescence of PI(4,5)P_2_ binding proteins, several of which are involved in actin remodeling (11). Therefore, overexpression of the catalytically dead form of PIP5Kβ prevents PI(4,5)P_2_ synthesis and the recruitment of actin remodeling complexes. On the other hand, overexpression of PIP5Kβ or -γ prevents the depletion of PI(4,5)P_2_ by hydrolysis from the PM and antagonizes vacuole formation.

Depletion of PI(4,5)P_2_ is critical for several endocytic processes, with its loss leading to uncoating of clathrin-coated vesicles during endocytosis or the clearance of F-actin during phagocytosis and macropinocytosis (12). During Y. pseudotuberculosis invasion, PI(4,5)P_2_ metabolism appears to be essential for membrane scission (64). PI(4,5)P_2_ is metabolized via multiple pathways, including (i) hydrolysis by phospholipase C to produce inositol 3,4,5-triphosphate and diacylglycerol, (ii) removal of 4′ and 5′ phosphates via inositol polyphosphate 4- or 5-phosphatases, and (iii) conversion to PI(3,4,5)P_3_ by PI3K (11). Previous work has demonstrated that inhibition of PI3Ks decreases R. conorii infection, suggesting that PI(3,4,5)P_3_ synthesis may be important in rickettsial entry (31). Using the PH domain of AKT, we observed an accumulation of PI(3,4,5)P_3_ around R. typhi during entry. Furthermore, inhibition of PI3Ks decreased infection, seemingly due to the inability to deplete PI(4,5)P_2_ from the PM via its phosphorylation to PI(3,4,5)P_3_. Following cup closure and scission from the PM, PI(3)P is a key marker and vacuole maturation determinant. Using tandem FYVE domains of EEA1 as a PI(3)P biosensor, we found PI(3)P completely surrounds the bacterium early during infection, but as infection progressed, PI(3)P was punctated around R. typhi. This corresponds with the conceptual model of rickettsial infection by which rickettsia transiently remains in a vacuole, which is then lysed by rickettsial phospholipases, allowing bacterial escape into host cytosol (70).

**FIG 7:**
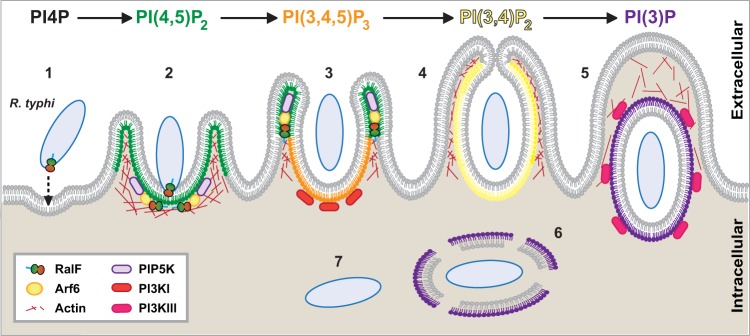
Schematic of PI metabolism during R. typhi entry. Upon engagement with host cells, we previously demonstrated that R. typhi secretes RalF via its type IV secretion system, resulting in recruitment of host Arf6 to PI(4,5)P_2_-enriched regions of the PM (48). Here we show that Arf6 is activated during R. typhi infection, leading to recruitment of PIP5K, which is critical for R. typhi infection (steps 1 to 3). Additionally, we established the downstream effects of this process by characterizing PI3K-mediated formation of the phagocytic cup and early endosome (steps 4 and 5). Following entry, R. typhi quickly escapes the phagocytic vacuole to reside within the cytoplasm of the host cell (steps 6 and 7).

Altogether our findings demonstrate that RalF_Rt_-activated Arf6 recruits PIP5K to the R. typhi entry foci to generate PI(4,5)P_2_, which presumably recruits actin remodeling proteins to promote cup formation. Removal of PI(4,5)P_2_ from the PM by PI3K is then predicted to allow for vacuole scission from the membrane. R. typhi transiently remains in a vacuole composed primarily of PI(3)P, with rickettsial phospholipases subsequently lysing the vacuole to allow bacterial access to the host cytoplasm (Fig. 7). Herein, our identification of a lineage-specific mechanism of rickettsial entry exemplifies the idea that different species of Rickettsia utilize lineage-specific factors to invade and colonize their host cells and identifies a previously unappreciated mechanism for host cell invasion.
